# Quantification of HER family receptors in breast cancer

**DOI:** 10.1186/s13058-015-0561-8

**Published:** 2015-04-09

**Authors:** Paolo Nuciforo, Nina Radosevic-Robin, Tony Ng, Maurizio Scaltriti

**Affiliations:** 1 Molecular Oncology Laboratory, Vall d’Hebron Institute of Oncology, Passeig Vall d’Hebron 119–129, Barcelona, 08035 Spain; 2 Universitat Autònoma de Barcelona, Barcelona, 08035 Spain; 3 ERTICa Research Group, University of Auvergne EA4677, 63000 Clermont-Ferrand, France; 4 Biopathology, Jean Perrin Comprehensive Cancer Center, 58 rue Montalembert, 63011 Clermont-Ferrand, France; 5 Richard Dimbleby Department of Cancer Research, Randall Division of Cell and Molecular Biophysics and Division of Cancer Studies, King’s College London, London, SE1 1UL UK; 6 UCL Cancer Institute, Paul O’Gorman Building, University College London, London, WC1E 6DD UK; 7 Breakthrough Breast Cancer Research Unit, Department of Research Oncology, Guy’s Hospital King’s College London School of Medicine, London, SE1 9RT UK; 8 Human Oncology and Pathogenesis Program (HOPP), Memorial Sloan Kettering Cancer Center, 1275 York Avenue, Box 20, New York, NY 10065 USA

## Abstract

The clinical success of trastuzumab in breast cancer taught us that appropriate tumor evaluation is mandatory for the correct identification of patients eligible for targeted therapies. Although HER2 protein expression by immunohistochemistry (IHC) and gene amplification by fluorescence *in situ* hybridization (FISH) assays are routinely used to select patients to receive trastuzumab, both assays only partially predict response to the drug. In the case of epidermal growth factor receptor (EGFR), the link between the presence of the receptor or its amplification and response to anti-EGFR therapies could not be demonstrated. Even less is known for HER3 and HER4, mainly due to lack of robust and validated assays detecting these proteins. It is becoming evident that, besides FISH and IHC, we need better assays to quantify HER receptors and categorize the patients for individualized treatments. Here, we present the current available methodologies to measure HER family receptors and discuss the clinical implications of target quantification.

**Electronic supplementary material:**

The online version of this article (doi:10.1186/s13058-015-0561-8) contains supplementary material, which is available to authorized users.

## Introduction

The HER family (also called ErbB or epidermal growth factor receptor (EGFR) family) comprises four transmembrane receptor tyrosine kinases, EGFR (or HER1), HER2, HER3, and HER4. These receptors signal through homo- and heterodimerization and promote cell proliferation, motility, and invasion [1]. Dysregulated expression and activity of HER family members is frequent in breast cancer. Overexpression of EGFR1, HER2 and HER3 is generally associated with poor prognosis whereas high expression of HER4 is associated with a better outcome [2-7]. Up to 25% of breast carcinomas overexpress HER2. High levels of this oncogene, almost invariably as a consequence of genomic amplification of a region of chromosome 17 (17q21) including the HER2 locus, drives aggressive disease and is an important therapeutic target.

Monoclonal antibodies (that is, trastuzumab, pertuzumab, T-DM1) and small molecule kinase inhibitors (that is, lapatinib, neratinib and afatinib) are the main strategies to target HER2 in breast cancer. Trastuzumab, in combination with chemotherapy, has significantly increased both progression-free survival (PFS) and overall survival in patients with advanced disease [8,9] as well as in the early (adjuvant) setting [10,11]. Lapatinib, given in combination with capecitabine, has shown clinical activity in HER2-positive breast cancer patients that became refractory to trastuzumab-based therapy [12]. Moreover, measurable clinical benefit is observed also when lapatinib is administered as a single agent or in combination with paclitaxel as first-line treatment [13,14]. Recently, the antitumor activity of dual HER2 blockade (trastuzumab in combination with either lapatinib or pertuzumab) was proven to be significantly superior to single agents in a neoadjuvant setting [15-17].

EGFR has been shown to be highly expressed in triple-negative breast cancer (TNBC), both in cell lines and in patients [18]. Moreover, preclinical studies have demonstrated that the inhibition of EGFR affects growth in TNBC cell lines [19]. These findings provided the rationale to test the efficacy of anti-EGFR agents, such as the antibodies cetuximab and panitumumab, in TNBC patients. In the metastatic setting, cetuximab in combination with chemotherapy showed some promising activity [20-22]. Nevertheless, no substantial improvements in either PFS or overall survival were achieved in these patients. A small pilot study testing the efficacy of panitumumab in combination with standard chemotherapy in TNBC patients in the neoadjuvant setting showed a pathological complete response rate of 46.8% [23]. However, the relevance of these findings will be assessed only when PFS and/or overall survival data are available.

There are several unanswered questions about which patients with breast cancer are most likely to benefit from one or another form of anti-HER targeted therapy and which type of determination methodology is most appropriate.

DNA-based, RNA-based, and protein-based assays have been developed to determine the HER status of breast tumors. To date, exploitation of the overexpression of HER2 is part of the management of a breast cancer patient whereas EGFR, HER3, and HER4 determinations are still exploratory and not used in clinical practice. As a matter of fact, to be eligible for anti-HER therapy such as trastuzumab, specimens have to be HER2-positive as determined by immunohistochemistry (IHC) analysis or harbor HER2/neu gene amplification by fluorescence *in situ* hybridization (FISH). Although these tests have become the benchmarks for defining tumors as HER2-positive, considerable controversy still exists regarding the accuracy, reliability, and inter-observer variability of these assay methods. It is estimated that up to 20% of HER2 testing performed in the field may be inaccurate when validated against central or 'expert' laboratories [24,25]. A recent round-robin study conducted to evaluate current HER2 testing methods and their potential impact on clinical outcomes showed that interpretation issues (especially when dealing with IHC or FISH equivocal results as defined by the American Society of Clinical Oncologists (ASCO)/College of American Pathologists (CAP) guidelines) and/or HER2 tumor heterogeneity may play a significant role in discordant results [26].

In an effort to improve the accuracy and consistency of HER2 testing, a joint task force of ASCO and CAP proposed guideline recommendations for HER2 testing using either IHC or FISH [27]. Among ‘HER2-positive’ tumors (defined by consensus criteria), there is a wide range of variability in terms of HER2-gene amplification and protein expression measured by conventional semi-quantitative methods such as the HercepTest®. The possibility that a quantitative analysis of HER family protein expression could improve the prediction of HER-targeting drugs has led to the evaluation of alternative and more quantitative tests. Despite that, the 2013 ASCO/CAP Update Committee concluded that there was insufficient evidence to warrant inclusion of these new assays to determine HER2 status in unselected patients due to lack of a consistent body of evidence on their analytical validity, clinical validity, and clinical utility [27].

In this review we address these issues by evaluating the current methodologies used for HER family status determination and discussing the clinical implications of HER family quantification on response to anti-HER treatment. In Additional file 1 we list the Food and Drug Administration (FDA) approved/Clinical Laboratory Improvement Amendments certified diagnostic tests available to measure HER receptors in the clinic.

## Methodologies

### HER status assessment at the protein level

#### Immunohistochemistry

IHC is the primary technique used to determine protein expression status in a patient sample. It is a simple, fast, easy to implement and relatively inexpensive method for protein detection. Slides are incubated with an antibody directed against the HER receptor protein, labeled, and finally made visible with a chromogen, resulting in a staining localized in the cellular compartment where the protein target is expressed (membrane, cytosol, nucleus). The more the protein is present, the stronger the staining will be. Traditionally, assessment of protein expression is done by visual estimation of staining intensity and is reported as binary (positive versus negative), four-tiered (0, 1+, 2+, and 3+), or semiquantitative continuous variable as for the H score ((% at 0) × 0 + (% at 1+) × 1 + (% at 2+) × 2 + (% at 3+) × 3; range = 0 to 300) results [28].

For companion diagnostic tests, guidelines are generally issued to guide pathologists in the interpretation and scoring of the staining. The HER2 scoring guidelines recommended by ASCO/CAP classified HER2 expression as 0 (no staining or faint incomplete membrane staining observed in ≤10% of tumor cells), 1+ (faint/barely perceptible incomplete membrane staining in >10% of tumor cells), 2+ (circumferential membrane staining that is incomplete and/or weak/moderate and within >10% of tumor cells or complete and circumferential membrane staining that is intense and within ≤10% of tumor cells) or 3+ (circumferential membrane staining that is complete, intense, and within >10% of tumor cells). Tumors with scores 0 and 1+ were considered negative; 2+ was considered equivocal and required FISH reflex testing; 3+ was considered positive and eligible for trastuzumab [29].

Despite the effort to standardize HER2 status determination, current guidelines do not restrict the type and characteristics of IHC assay to be used for HER2 protein expression. The use of FDA approved tests such as HercepTest® (DAKO, Carpinteria, CA, USA), PATHWAY anti-HER-2/neu (Ventana Medical systems, Roche, Tuscon, AZ, USA), InSite™ Her-2/neu (Biogenex, Freemont, CA, USA) as well as fully automated staining systems such as Ventana Benchmark (Ventana Medical systems, Roche, Tuscon, AZ, USA) and Leica Microsystems Bondmax (Leica, Newcastle, UK) may certainly minimize process variability and improve assay repeatability and reproducibility. Nevertheless, many laboratories developed tests with different antibodies directed against other HER2 epitopes (intracellular versus extracellular), which may show non-overlapping specificities and be differently influenced by preanalytical factors [30-32].

Interpretation of membrane staining can be optimized using quantitative image analysis such as the automated quantitative analysis (AQUA) system [33]. AQUA is a fluorescence IHC-based method that provides objective and continuous protein expression scores for tissues by using automated fluorescence microscopy and advanced image analysis algorithms. It is important to note, however, that there are as yet no clinical data related to AQUA’s predictive ability.

Other software applications include Aperio Scanscope (Aperio Technologies; Vista, CA, USA), Definiens (Carlsbad, CA, USA) and Vysis AutoVysion (Abbott Molecular, Des Plaines, IL, USA) among others. These applications can reduce the subjectivity of a traditional scoring system and provide a more reproducible protein expression score [34,35].

#### Enzyme-linked immunosorbent assay

HER2 receptor protein extracellular domain (ECD, p105) is released into the circulation after cleavage by matrix metalloproteinases and its levels can be measured in the serum using an enzyme-linked immunosorbent assay approved by the FDA (Siemens Healthcare, Erlangen, Germany). Elevated levels of serum HER2 ECD have been shown to be both prognostic and predictive of response to trastuzumab in HER2-positive tumors [36,37]. Serum ECD values have been suggested, therefore, as an alternative technique for determining HER2 status, although available results are controversial. First, not all patients with HER2-positive tumors appear to have elevated serum ECD values and patients with HER2-negative tumors can also have elevated ECD values. Second, the reported data come from studies including a limited number of patients, thus making current evidences still insufficient to consider basing treatment decisions on ECD levels in routine clinical practice. A large meta-analysis study [38] has combined the data of four trastuzumab trials in metastatic breast cancer and showed that, from the combined dataset (N = 322 patients), there was no correlation between baseline ECD value and tumor response. ECD values decreased upon initiation of combination therapy irrespective of treatment and tumor response. Furthermore, disease progression was not reliably predicted by an increase in ECD levels. Therefore, the use of ECD values in treatment decision making was not recommended.

#### VeraTag™ proximity-based assay

The VeraTag™ proximity-based assay (HERmark® Breast Cancer Assay; Monogram Biosciences, Inc., South San Francisco, CA, USA) enables precise quantitative measurements of total HER-2 expression and HER2 homodimers in formalin-fixed, paraffin-embedded (FFPE) tissue specimens [39,40]. The HERmark assay was developed based on a proprietary proximity-based technology platform that enables accurate quantification of proteins and protein-protein complexes through the release of a fluorescent tag (VeraTag reporter, Monogram Biosciences) conjugated to a pair of monoclonal antibodies directed to unique epitopes on the HER2 receptor in molecular proximity [40]. The continuous total HER-2 expression results are grouped as HERmark negative, HERmark equivocal, and HERmark positive. The threshold for a positive HERmark test is based on the comparison with HER2 tests performed in 1,090 breast tumor reference samples (central IHC and central *in situ* hybridization) from three different study cohorts. The HERmark assay can detect HER2 at amounts of 2,500 up to more than 1 million receptors per cell, and is thus said to be 7 to 10 times more sensitive than IHC. The assay has been validated according to the specifications prescribed by the Clinical Laboratory Improvement Amendments and is performed only in the CAP-certified clinical reference laboratory at Monogram Biosciences (US). VeraTag™ proximity-based assays have been developed also to measure total EGFR, EGFR-EGFR homodimers and EGFR-HER2 heterodimers [40-42], p95 [43], total HER3, HER3-HER3 homodimers and HER3-phosphoinositide 3-kinase (PI3K) complex heterodimers [44] and the phosphorylated forms of EGFR, HER2, and HER3.

#### Protein interaction measurements

Clinical application of protein-protein interactions has uncovered many potential targets for novel drug development or drug resistance mechanisms [45], with the MDM2-p53 interaction [46,47] and B-Raf inhibition being examples of recent successes [48]. More recently, incorporation of protein interaction data was shown to also improve the predictive performance of prognostic gene expression signatures [49,50]. Despite the importance of adjunct information supplied by the protein interactome configuration to improve the existing prognostic signatures for predicting patient outcome [50], this protein interaction information has rarely been incorporated in diagnostic/prognostic assays.

Fluorescence lifetime imaging microscopy (FLIM) is based on quantifying the non-radiative transfer of energy between the donor and acceptor fluorophores and can only occur when the two molecules are no further apart than 10 nm, consistent with being in molecular contact [51-53]. Various automated imaging platforms, including ours, measure Förster resonance energy transfer (FRET) - the decrease in donor lifetime, the gold standard for FRET measurements (reviewed recently in [54]) - to directly monitor validated protein-protein interactions [55-61] and post-translational modifications, including conformational changes, in cultured cells [58,62-66]. A two antibody FRET/FLIM approach was originally applied, by ourselves and others, to human cancer tissues to detect the nano-proximity between a donor fluorophore-conjugated anti-protein kinase C or anti-EGFR antibody, and an acceptor fluorophore-labeled phospho-specific antibody, providing highly specific quantification of phosphorylation [67,68]. Detailed methodology for sample preparation and instrumentation can be found elsewhere [69,70]. We have now extended this method to measure endogenous protein-protein interactions in archived pathological material [71]. The presence of autofluorescence in stromal and epithelial components may cause difficulties in accurately determining the fluorescence lifetime of fluorophores in FFPE tissue samples [72]. By circumventing the autofluorescence issue using a new analysis algorithm [73], we have recently described the first clinical utilization of this refined FLIM assay (using Alexa546 and Cy5 as donor and acceptor fluorophores, respectively) to quantify the level of HER1-HER3 dimer formation in FFPE tissues from basal-like breast cancer patients who were treated with a neoadjuvant anti-EGFR treatment (cetuximab or panitumumab) [74]. Moreover, we have demonstrated the existence of EGFR-HER4 dimers in breast cancer cells and how these dimers are important for cell motility [75].

Liquid chromatography-tandem mass spectrometry-based proteomics has emerged as the most effective method to study complex proteomes. In this approach, the proteins representing a proteome are analyzed after enzymatic digestion by liquid chromatography coupled to mass spectrometry (MS). Although this approach is a powerful tool to identify proteins in complex biological samples [76,77], it is not optimal for systematic quantification of these proteins because of the stochastic nature and the limited sensitivity of the approach. During the past few years, targeted proteomics has been shown to be complementary to the more widely used discovery proteomic methods. In targeted proteomics, only pre-determined peptide ions are selected for detection and quantification in a sample. The main MS approach supporting targeted proteomics is selected reaction monitoring (SRM), where specific MS assays are generated *a priori* and used to selectively detect and quantify proteins of interest in a sample. This approach can provide objective quantification and multiplex capabilities with high sensitivity and in an antibody-free setting [78-80]. SRM methods have long been used to quantify low-abundance protein targets in plasma [81] but application of these techniques to FFPE tissue samples has, until recently, been hindered by incomplete solubilization of samples [82,83]. The Liquid Tissue-(SRM) diagnostic technology platform is a newly developed proteomic method that overcomes this limitation, allowing for precise protein quantification in FFPE tissues. Microdissected FFPE tumor tissues are subjected to Liquid Tissue processing to reverse formalin crosslinks. This is followed by trypsinization to completely solubilize all of the protein in the sample. This tryptic peptide mixture is then subjected to SRM analysis using stable isotope-labeled control peptides for accurate quantification [83-85]. Multiple reports have demonstrated that comparable results may be obtained between formalin fixed and matching frozen tissue [84,86]. The reliability of this approach for analysis of proteins in any biological sample including FFPE patient tumor tissue has been demonstrated [87-91], thus widening the application of MS to patient-derived tissue with a consequent profound impact on patient stratification and targeted cancer therapeutics.

Reverse phase protein array (RPPA) and collaborative enzyme enhanced reactive-immunoassay (CEER) are nano-scaled dot blot platforms allowing the detection of multiple proteins (both total and phosphorylated) in many samples simultaneously. They do not require large amounts of sample but are not suitable for FFPE tissue. For RPPA protein lysates are immobilized onto microarrays and then probed with the primary antibodies of choice. Detection is performed by quantification of the labels (fluorescent, colorimetric or other kinds) bound to either the primary or, more often, the secondary antibody added to amplify the signal. RPPA allows testing hundreds of samples at the same time and multiplexing is performed by analyzing multiple arrays spotted with the same protein lysates with different antibodies [92].

CEER takes advantage of the immunocomplexes formed between antibodies printed on a nitrocellulose microarray surface with the target molecules in cell lysates. Once the complexes are formed, two detector antibodies (one conjugated to glucose oxidase and another conjugated to horse radish peroxidase (HRP)) are added. Target detection (expressed as computational units (CU)) requires the presence of both detector antibodies, and the enzyme channeling event between glucose oxidase and HRP will not occur unless both antibodies are in close proximity [93]. The main difference with RPPA is that, instead of protein lysate, antibodies are immobilized in cellulose arrays. This means that, contrary to RPPA, CEER is capable of measuring the expression of dozens of targets simultaneously in the same sample.

Further studies are needed to prove the clinical relevance of the above described methods.

### HER status assessment at the DNA level

#### *In situ* hybridization

FISH is considered the gold standard method for gene amplification status determination. FISH uses fluorescently labeled probes (usually red) that are complementary to a part of the target gene. After hybridization to the complementary DNA on the slide, the probes can be visualized with a fluorescence microscope. A second probe labeled with a different fluorochrome (usually green) directed against the centromeric region of the chromosome containing the target gene is generally used as control for polysomy. The number of copies of the target gene and centromere probe can be estimated and the ratio determined.

CISH (chromogenic *in situ* hybridization) is an alternative for FISH. It uses an immunoperoxidase reaction to visualize the target gene probe, which allows scoring with a conventional light microscope. CISH has several advantages over FISH: signal does not fade and the slides can be kept permanently and allows better preservation of morphology. One of the main limitations of CISH is that most of the available assays are still monoprobe assays, meaning that there is no correction with a centromere control probe and only the absolute gene copy number is scored.

Similar to CISH, silver *in situ* hybridization (SISH) technology uses a non-fluorescent method where the HRP bound to the probe catalyses the reduction of silver acetate to produce a black signal. Several studies showed a good correlation between FISH, CISH, SISH, and IHC for HER2 status determination [94-100].

Recently released ASCO/CAP guidelines recommended that HER2 must be considered *in situ* hybridization (ISH)-positive based on a single-probe average HER2 copy number ≥6.0 signals/cell or dual-probe HER2/CEP17 ratio ≥2.0 or dual-probe HER2/CEP17 ratio <2.0 with an average HER2 copy number ≥6.0 signals/cell [27]. Whether the centromere control probes for polysomy 17 are really necessary is a matter of debate given that it has been proven by several studies that true polysomy 17 is very rare in breast carcinomas [101]. Concurrent evaluation of several chromosome 17 genes using multiple-probe FISH or multiplex ligation-dependent probe amplification showed that focal amplifications encompassing the centromere - and not true polysomy - are the most common explanation for increases in CEP17 signals [102,103]. These results suggest that CEP17 copy number assessment by standard ISH is not a useful surrogate for polysomy 17. Compared with IHC, ISH assays, in which the target gene copy number is counted, are considered to be more quantitative analytically. However, ISH is not a direct measurement of the protein and just because a change in gene copy number is observed does not necessarily mean that it is expressed. In addition, the procedure is time consuming and new 'fast' FISH assays are under development to reduce the turnaround time [104].

#### PCR-based techniques

PCR-based techniques such as multiplex ligation-dependent probe amplification [105] have several advantages over ISH-based assays. First, they are more quantitative and results are easier to interpret. Second, they require only small amounts of DNA and are not affected by DNA degradation, thus performing well with FFPE samples. Third, they can be multiplexed, allowing simultaneous interrogation of multiple genes or different parts of genes, representing an ideal and low cost prescreening tool. Head-to-head comparisons between IHC, FISH, and CISH have shown good correlation among technologies [106-109]. The main weaknesses of PCR-based assays are that they do not preserve tissue morphology, may require sample macro- or microdissection to enrich for tumor content, heterogeneity can be missed and contamination with normal or ductal carcinoma *in situ* may lead to both false-negative and false-positive results.

### HER status assessment at the RNA level

Due to multiplexing capability, RNA-based tests are usually used to generate global gene expression signatures rather than single gene measurements. All these signatures work using proprietary algorithms that generate a score based on the expression levels of the genes measured that can determine risk factors, incidence, prognoses and responses to systemic therapies. Clinically validated gene expression tests that include one or more HER family members in their gene lists are discussed below.

The Onco*type* DX assay (Genomic Health, Redwood City, CA, USA) uses RT-PCR as a primary technique and work on RNA extracted from FFPE samples. The assay measures the expression of a panel of 21 genes (only *HER2* is included among the HER family genes) and the results are provided as a recurrence score. Although the assay was approved as a prognostic test predictive of breast cancer recurrence in women with newly diagnosed, early stage breast cancer, it also assesses the benefit from certain types of chemotherapy [110]. Recently, Genomic Health started reporting estrogen receptor (ER), progesterone receptor (PR), and HER2 results separately in addition to the recurrence score. Although high overall concordance (greater than 91%) between HER2 by IHC or FISH assay and quantitative RT-PCR using the Onco*type* DX test has been reported [111,112], an independent study showed a false-negative rate for Onco*type* DX RT-PCR for HER2 of >50% [113].

TargetPrint™ (Agendia, Irvine, CA, USA/Amsterdam, The Netherlands) is a microarray-based gene expression test that allows quantitative assessment of ER, PR and HER2 at the RNA level in breast cancer. Compared with IHC results, HER2 gene expression levels provided by TargetPrint™ have been shown to be more reproducible and truly quantitative. Results were validated against IHC and showed an overall concordance greater that 95% [114-116]. Its use is currently proposed in case of equivocal or unreliable IHC results, discordance between two separate tests, discordance of test results and clinicopathologic features or technical failure of IHC/FISH/CISH.

The NanoString Prosigna™ (NanoString Technologies, Seattle, WA, USA) assay measures the expression levels of 50 target genes (including HER2) plus eight constitutively expressed normalization genes (PAM50) to classify a tumor as one of four intrinsic subtypes (luminal A, luminal B, HER2-enriched, and basal-like), which have been shown to be prognostic [117,118]. In addition to identifying a tumor’s intrinsic subtype, the PAM50 signature generates an individualized score estimating a patient’s probability of disease recurrence by weighting the molecular subtype correlations, a subset of proliferation genes, and pathologic tumor size [118,119]. Based on these data, the FDA-cleared and CE-marked Prosigna™ assay, based on the PAM50 gene expression signature, has recently been shown to predict the risk of distant recurrence in women with hormone receptor-positive early stage breast cancer treated with 5 years of endocrine therapy [120-122]. The Nanostring nCounter system uses color-coded probes that bind directly to the RNA transcript without reverse transcription and PCR amplification [123] and work in frozen or FFPE tissues with equivalent ease and efficiency [124]. Assay controls are included to ensure that test samples and the test process meet pre-defined quality thresholds.

The PAM50 gene signature may be run also by classic quantitative PCR and can also provide quantitative and qualitative gene expression scores for the standard biomarkers usually measured semi-quantitatively by IHC - ER, PR and HER2. Using the quantitative PCR cutoff for ERBB2 expression, a study found high specificity (609/624 samples that were low ERBB2 were also HER2-negative by IHC/CISH), while 53% (109/190) of tumors with intermediate-high ERBB2 expression were HER2-positive [125]. This same study and the MA.5 trial [126] found that only about two-thirds of clinically HER2-positive tumors are classified as HER2-enriched. Thus, only a subset of the IHC-defined groups overlap with PAM50 subtype classification.

Although some literature shows an overall high concordance between standard techniques such as IHC and FISH assays and quantitative RT-PCR [111,127-129], there are several practical issues that should be considered when conducting RNA-based analyses. First, the presence of normal tissue within the tumor sample is a major source of subtype misclassification [130]. Therefore, identification of the region of viable invasive breast carcinoma by a pathologist is critical before any RNA extraction is performed. Second, RNA shows greater instability compared with DNA and proteins and thus the selection of technologies that may prevent/overcome RNA degradation is important.

## Clinical implications

The fundamental principle of targeted therapy is to specifically harm tumor cells that depend on a definite target for proliferation and survival, sparing non-tumor cells from damage. In many cases, the target is a protein with activating mutations that is present only in tumor cells, facilitating the specificity of the therapy (for example, Braf-mutant melanomas, EGFR-mutant lung cancer), allowing profound inhibition of the target before the emergence of side effects. In the case of HER receptors in breast cancer the target is a protein that, although not carrying any activating alterations, is present in much higher amounts in tumor cells compared with normal cells. In these cases one would guess that the higher is the difference in target expression between normal and tumor cells, the wider is the therapeutic window. However, only the presence of the target or its semiquantitative expression (and not the absolute levels) is currently taken into consideration in clinical practice.

There is an increasing body of evidence indicating that the levels of HER2 in HER2-positive tumors can influence the response to HER2-targeted therapy, converging to the common conclusion that 'more HER2, more response' [131-137]. Quantitative HER2 expression or homodimer levels determined by the HERmark assay correlated with clinical outcome of trastuzumab therapy better than IHC or central FISH studies in patients with metastatic breast cancer. Interestingly, patients with *HER2* gene amplification by FISH but low HER2 protein expression or homodimer levels as measured by HERmark responded poorly to trastuzumab-containing therapy, suggesting that not all gene-amplified tumors overexpress the target of trastuzumab [135]. Similarly, absolute HER2 quantification in an homogeneous group of HER2-positive breast cancer (IHC 3+) using triple quadrupole MS was predictive of a better response to trastuzumab in both adjuvant and metastatic settings [136].

But perhaps this is valid until a certain limit. First, the link between the level of HER2 amplification and outcome in patients treated with trastuzumab has been proven only in the neoadjuvant setting [138], whereas other studies failed to demonstrate this association [139,140]. Second, although the clinical benefit from HER2 blockade increases with the level of the target, there may be tumors with extraordinarily high levels of HER2 that are actually more resistant to the therapeutic pressure [141-144]. It is unclear whether this is due to insufficient engagement of the receptor by the targeted agents. In any case, validation of these findings in a larger cohort of patients is necessary. Third, the intriguing observation from B-31 and N9831 studies that tumors that failed to be confirmed as HER2-positive after central laboratory testing may still derive benefit from trastuzumab [145,146] and the complex relationship between HER2, ER, and trastuzumab sensitivity outlined by the study suggest that quantitative HER2 measure alone may not be sufficient, and combination with other markers may be more predictive of trastuzumab response [147].

Since dual HER2 blockade (trastuzumab combined with either pertuzumab or lapatinib) is proving to be more effective than single agent treatment, it will be interesting to investigate whether HER2 absolute levels predict response in this setting as well. In the neoadjuvant setting, this seems to be the case. HER2 levels were measured by HERmark in the primary tumors of patients enrolled in the NeoALTTO trial, testing the activity of trastuzumab in combination with lapatinib compared with single agent treatments, and a positive correlation was found between constitutive HER2 expression and benefit from dual blockade [148].

One of the mechanisms proposed for the synergy observed when combining lapatinib and trastuzumab (at least in preclinical models) is the stabilization and membrane accumulation of HER2 as a consequence of receptor kinase inhibition [149]. One may wonder, therefore, whether lapatinib could sensitize tumors with relatively low levels of HER2 to the antitumor activity of trastuzumab. Testing this possibility, however, is not as easy as it sounds. First, a threshold above which tumors benefit from anti-HER2 therapy (but are still considered 'low expressing tumors') needs to be defined by quantitative methodology. Then, other therapeutic options should be considered to exclude the possibility that these patients can achieve better response from other agents. Genomic analysis of the tumors would be very helpful in these cases as the identification of actionable genetic alterations may guide the choice of therapy. Finally, HER-targeted therapeutic agents such as lapatinib have been shown to stabilize/enhance the HER2-HER3 dimer in preclinical cell models [149]. The quantification of this dimer (as described above), which is believed to be the most potent of all HER dimers with regard to driving cellular proliferation [150,151], will provide important and non-redundant information to that provided by HER protein expression to help clinicians to understand and/or predict the heterogeneity in clinical response.

The quantification of HER3 in response to lapatinib-containing therapies may also be of relevance. In fact, compensatory upregulation of HER3 upon lapatinib treatment has been described both in preclinical models and in patients with HER2-positive breast cancer [152]. The addition of compounds blocking HER3 or the downstream PI3K/AKT pathway significantly potentiates the antitumor effects of lapatinib, underscoring the importance of this occurrence. Because of the mechanistic relationship between EGFR and HER2, EGFR measurement may provide a method for personalizing treatment in breast cancer, beyond the single assay for HER2. Patients with high EGFR using the EGFR antibody D38B1 did not appear to benefit from concurrent trastuzumab in the N9831 trial using the fluorescence-based AQUA quantitative platform [153]. Based on these results, it may be hypothesized that the subset of tumors with high EGFR expression may better respond to lapatinib or dual HER blockade compared with trastuzumab alone.

The absolute levels of EGFR may be predictive for response to anti-EGFR therapy in TNBC patients. We recently showed that patients with tumors expressing high levels of EGFR were more likely to achieve pathological complete response following panitumumab-based therapy [74]. Furthermore, we found that EGFR levels tended to decrease in the residual tumors collected at surgery compared with the primary tumor before the commencement of therapy, indicating that the levels of EGFR may be influenced by the therapeutic pressure. It remains to be defined whether this is a global downregulation of EGFR in all tumor cells or is a positive selection of cells with lower EGFR expression.

As a matter of fact, the acquired loss of expression of HER receptors may be an obvious mechanism of resistance to targeted therapy according to the simple paradigm 'no target, no response'. This has also been described in HER2-positive breast cancer patients upon treatment with trastuzumab-based therapy [154]. Therefore, measuring the levels of HER receptors at the time of progression to targeted therapy should be encouraged to avoid persevering with similar targeted approaches.

## Conclusion and perspectives

It is becoming evident that the 'simple detection' of the HER receptors in breast cancer is not sufficient to predict the benefit that patients will achieve from anti-HER therapy. The example of HER2 is archetypal. We know that HER2-positive patients benefit from anti-HER2 therapy, but now we also know that 15 to 20% of these patients express levels of the receptors that are almost comparable with HER2-negative tumors. And, more importantly, these patients do not achieve the same benefit from anti-HER2 therapy as do patients with high HER2 expression. This is especially true in the neoadjuvant setting in patients undergoing dual HER2 blockade [148].

Let’s make an example of how relevant these findings can be. The disease-free survival data from the ALTTO adjuvant trial (comparing patients that received lapatinib, trastuzumab or the combination of the two agents) were recently released [155]. The take home message was that the combination was not significantly superior to trastuzumab single agent in preventing relapses to therapy. These findings were somehow surprising since the NeoALTTO trial clearly demonstrated that dual HER2 blockade is more effective than monotherapy in the neoadjuvant setting. But if we dissect the data we realize that many variables could have influenced this outcome. First, the number of PFS events taken into consideration was lower than the one needed for the planned statistical analysis. Second, a significant percentage of patients enrolled in the combination arm were not treated with a full dose of lapatinib (for toxicity reasons). In a study where the 'control arm' (trastuzumab-based therapy) is known to cure more than 80% of patients, these factors may have diluted the possible improvement in PFS. Thus, it is not so surprising that the difference observed in the ALTTO trial was not significant. It would be interesting to quantify the levels of HER2 in these samples and correlate them with clinical response. Perhaps we will identify a subset of patients with high HER2 expression that is more sensitive to dual HER2 blockade and shows significant clinical benefit in the long term. Fortunately, these samples are available for future biomarker analyses, including HER2 quantification.

For EGFR things are far behind. The basis for testing anti-EGFR therapy in TNBC was the knowledge that overexpression of EGFR occurs in up to 50% of cases [156]. But a real stratification based on how much EGFR these tumors express has never been made. Now we have evidence that, the higher the levels of EGFR, the higher the probability to achieve pathological complete response from cetuximab- or panitumumab-based therapy in the neoadjuvant setting [74]. Again, one would wonder whether the reported activity of anti-EGFR therapy in TNBC (or even in head and neck and colon cancers) would be different if stratification based on the EGFR levels had been done in these clinical trials.

## Conclusion

In conclusion, HER receptor quantification may be more tedious than FISH or IHC but it can help in stratifying and selecting patients for anti-HER therapy. Measuring the levels of the targets in patients undergoing 'targeted' therapy sounds like a good idea.

## Additional file

Additional file 1:
**A table listing laboratory diagnostic tests cleared by the Food and Drug Administration or offered by central laboratories under Clinical Laboratory Improvement Amendments measuring HER receptors in the clinic.** *Epidermal growth factor (EGFR), HER2 and HER3. ^#^Approved for colorectal cancer. LDT, laboratory developed test; Q, quantitative; QL, qualitative; SQ, semiquantitative.

## Abbreviations

AQUA: Automated quantitative analysis
ASCO: American Society of Clinical Oncologists
CAP: College of American Pathologists
CEER: Collaborative enzyme enhanced reactive-immunoassay
CISH: Chromogenic *in situ* hybridization
ECD: Extracellular domain
EGFR: Epidermal growth factor receptor
ER: Estrogen receptor
FDA: Food and Drug Administration
FFPE: Formalin-fixed, paraffin-embedded
FISH: Fluorescence *in situ* hybridization
FLIM: Fluorescence lifetime imaging microscopy
FRET: Förster resonance energy transfer
HRP: Horse radish peroxidase
IHC: Immunohistochemistry
ISH: *In situ* hybridization
MS: Mass spectrometry
PFS: Progression-free survival
PI3K: Phosphoinositide 3-kinase
PR: Progesterone receptor
RPPA: Reverse phase protein array
SISH: Silver *in situ* hybridization
SRM: Selected reaction monitoring
TNBC: Triple-negative breast cancer
