# Supplementary material for: Quantification of HER family receptors in breast cancer
Source: Breast Cancer Res. 2015 Apr 9;17:53. doi: 10.1186/s13058-015-0561-8 (PMC4389676; doi:10.1186/s13058-015-0561-8)
Supplement: Additional file 1: — A table listing laboratory diagnostic tests cleared by the Food and Drug Administration or offered by central laboratories under Clinical Laboratory Improvement Amendments measuring HER receptors in the clinic. *Epidermal growth factor (EGFR), HER2 and HER3. #Approved for colorectal cancer. LDT, laboratory developed test; Q, quantitative; QL, qualitative; SQ, semiquantitative. [file 13058_2015_561_MOESM1_ESM.doc]

| **Assay name** | **Provider** | **Method** | **Measurement** | **Receptor** | **Type of test** |
| --- | --- | --- | --- | --- | --- |
| InSite HER2/neu CB11 | Biogenex | IHC | total protein (SQ) | HER2 | FDA-approved |
| HERCEPTEST™ | DAKO | IHC | total protein (SQ) | HER2 | FDA-approved |
| HERmark™ | Monogram Bioscience | Proximity based | total protein, dimer (Q) | HER2 | LDT-CLIA |
| ADVIA Centaur® Serum HER-2/neu | Siemens Healthcare | ELISA | total protein (Q) | HER2 | FDA-approved |
| Pathway® Anti-HER2 (clone CB11) | Ventana-Roche | IHC | total protein (SQ) | HER2 | FDA-approved |
| Oncoplex Breast Cancer Proteomic Panel | Oncoplex Dx | Proteomics | total protein (Q) | HERs* | LDT-CLIA |
| EGFR pharmDx™ | DAKO | IHC | total protein (QL) | EGFR | FDA-approved^#^ |
| HER2 CISH pharmDx™ | DAKO | CISH | gene amplification (Q) | HER2 | FDA-approved |
| HER2 FISH pharmDx™ | DAKO | FISH | gene amplification (Q) | HER2 | FDA-approved |
| SPOT-Light® HER2 CISH | Invitrogen | CISH | gene amplification (Q) | HER2 | FDA-approved |
| Inform™ HER2 Dual ISH | Ventana-Roche | SISH | gene amplification (Q) | HER2 | FDA-approved |
| PathVysion® | Vysis (Abbott) | FISH | gene amplification (Q) | HER2 | FDA-approved |
| Oncotype DX® | Genomic Health | RT-PCR | gene expression (Q) | HER2 | LDT-CLIA |
| TargetPrint™ | Agendia | Microarray | gene expression (Q) | HER2 | LDT-CLIA |

**Table 1.** Laboratory diagnostic tests cleared by the FDA or offered by central laboratories under CLIA measuring HER receptors in the clinic.

SQ: semiquantitative; Q: quantitative; QL: qualitative; *EGFR, HER2 and HER3; ^#^Approved for colorectal cancer.
